# Disrupted neuronal trafficking in amyotrophic lateral sclerosis

**DOI:** 10.1007/s00401-019-01964-7

**Published:** 2019-02-05

**Authors:** Katja Burk, R. Jeroen Pasterkamp

**Affiliations:** 1 Department of Neurologie, Universitätsmedizin Göttingen, Robert-Koch-Str. 40, 37075 Göttingen, Germany; 2 Center for Biostructural Imaging of Neurodegeneration, Von-Siebold-Str. 3A, 37075 Göttingen, Germany; 3 Department of Translational Neuroscience, UMC Utrecht Brain Center, University Medical Center Utrecht, Utrecht University, Universiteitsweg 100, 3584 CG Utrecht, The Netherlands

**Keywords:** Amyotrophic lateral sclerosis, Motor neuron, Trafficking, Cytoskeleton, Rab

## Abstract

Amyotrophic lateral sclerosis (ALS) is a progressive, adult-onset neurodegenerative disease caused by degeneration of motor neurons in the brain and spinal cord leading to muscle weakness. Median survival after symptom onset in patients is 3–5 years and no effective therapies are available to treat or cure ALS. Therefore, further insight is needed into the molecular and cellular mechanisms that cause motor neuron degeneration and ALS. Different ALS disease mechanisms have been identified and recent evidence supports a prominent role for defects in intracellular transport. Several different ALS-causing gene mutations (e.g., in *FUS*, *TDP*-*43,* or *C9ORF72*) have been linked to defects in neuronal trafficking and a picture is emerging on how these defects may trigger disease. This review summarizes and discusses these recent findings. An overview of how endosomal and receptor trafficking are affected in ALS is followed by a description on dysregulated autophagy and ER/Golgi trafficking. Finally, changes in axonal transport and nucleocytoplasmic transport are discussed. Further insight into intracellular trafficking defects in ALS will deepen our understanding of ALS pathogenesis and will provide novel avenues for therapeutic intervention.

## Introduction

Amyotrophic lateral sclerosis (ALS) is a fatal disease characterized by the degeneration of upper and lower motor neurons causing muscle denervation. In the majority of patients, the cause of the disease is unknown and these cases are referred to as sporadic ALS (SALS) cases. In 5–10% of cases, there is a family history of ALS (FALS) [142]. The prevalence of ALS in most countries is around five cases per 100,000 people [2] with a median age of onset of SALS of 65 years, while, for genetically heterogeneous populations, onset is about 10 years earlier [44]. As disease progresses, corticospinal motor neurons, projecting from the motor cortex to the brainstem and spinal cord, and bulbar and spinal motor neurons, projecting to skeletal muscles, degenerate. Consequently, muscles innervated by these neurons deteriorate and patients usually die from respiratory failure within 3–5 years after symptom onset [44].

Despite the general notion that ALS is a neuromuscular disease, in many patients, the CNS is affected more generally. Between 5 and 15% of patients with ALS also have frontotemporal dementia (FTD), while up to 50% of ALS patients display cognitive or behavioral changes within the spectrum of FTD [44]. The mechanisms that cause motor neuron degeneration and ALS remain incompletely understood. Mutations in > 30 genes have been linked to FALS, and on basis of the functions of these genes, different disease pathways have been proposed and investigated. For example, in about 60–80% of patients with FALS, the most common mutations are in *C9ORF72* (40%), *SOD1* (20%), *FUS* (1–5%), and *TARBDP* (1–5%) [142]. These genetic defects suggest changes in molecular pathways controlling; for example, RNA biology, protein turnover, and axonal transport [144].

Interestingly, an increasing number of recent studies report defects in intracellular trafficking in ALS, but much remains unclear about the role of altered trafficking in motor neuron degeneration. For example, what is the precise effect of gene mutations on protein function and distribution? Do different affected proteins control separate steps of intracellular trafficking or does their function converge onto common pathways? In this review, we discuss different intracellular trafficking processes that have been linked to the pathogenesis of ALS. These range from endosomal trafficking and autophagy to axonal and nucleocytoplasmic transport. We discuss how these processes, and the proteins that control them, are altered in ALS and provide directions for future research.

## Disrupted receptor and endosomal trafficking

An increasing number of trafficking defects are being linked to the pathogenesis of ALS. In this section, we will discuss the evidence for changes in receptor and endosomal trafficking. In this and each of the following sections, the effects of individual ALS-associated genes are highlighted first, followed by a discussion on how these individual defects may be interconnected. When trafficking defects have been covered extensively in recent review articles, we will refer to these reviews and focus on the most significant findings.

One of the most impactful recent genetic findings in ALS is the discovery of an ALS-FTD causative mutation in Chromosome 9 open reading frame 72 (C9ORF72) in the form of a GGGGCC hexanucleotide repeat expansion in the first intron of the *C9ORF72* locus (from a typical 5–10 repeats in controls to hundreds or more in patients) [33, 136, 143, 177]. This mutation occurs with high frequency in individuals of European descent but less in other populations [76]. In humans, three alternatively spliced C9ORF72 transcripts exist, predicted to produce two polypeptide isoforms [33]. Different mechanisms have been proposed through which C9ORF72 repeat expansions contribute to ALS pathology. First, the hexanucleotide repeat expansion leads to genetic haploinsufficiency by forming stable G-quadruplex structures that disrupt transcription [50]. The repeat expansion may also promote hypermethylation of the locus, thereby further attenuating C9ORF72 expression [190]. Second, GGGGCC repeat-containing RNA accumulates in nuclear foci [33, 58] which may lead to toxic gain of RNA function through sequestration of RNA-binding proteins [170]. Third, GGGGCC repeat-containing RNA can undergo repeat-associated non-ATG (RAN) translation resulting in the generation of toxic dipeptide repeat (DPR) proteins which accumulate in the brain in disease [118, 119].

**Fig. 1:**
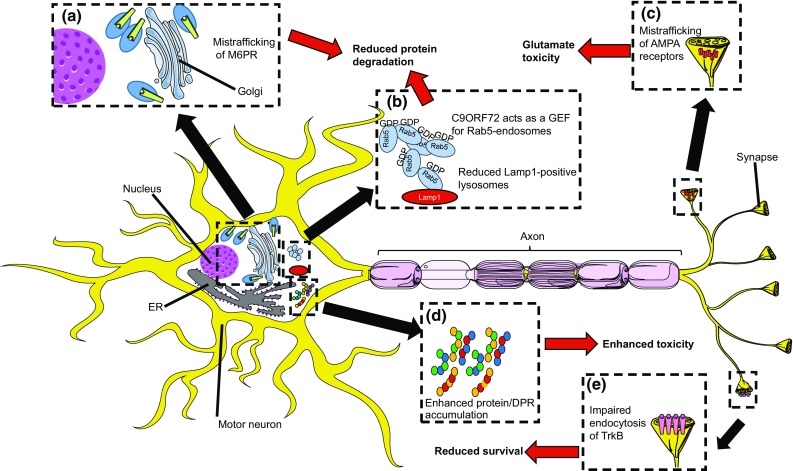
Effects of ALS-associated C9ORF72 repeat expansions. C9ORF72 hexanucleotide repeat expansions lead to C9ORF72 haploinsufficiency, and RNA and dipeptide repeat protein (DPR)-mediated toxic gain of function mechanisms that affect motor neurons (MNs) by deregulating endosomal and receptor trafficking leading to reduced protein degradation and enhanced aggregation, reduced survival, and glutamate toxicity. For several of these defects, it is unknown if they are caused by C9ORF72 loss and/or gain of function phenotypes. Only, for defects where evidence is compelling enough the precise mechanism (C9ORF72 loss or gain of function) is mentioned below. **a** M6P receptors (M6PR) are transported at slower rates and display subcellular mislocalization in C9ORF72 patient-derived induced (i)MNs. Since M6PR contributes to protein degradation by delivering cargo to lysosomes, M6PR mistrafficking may cause reduced protein degradation. **b** In MNs, C9ORF72 localizes to Rab5-positive early endosomes and acts as a Rab-GEF. In iMNs from C9ORF72 ALS patients and in spinal motor neurons in *Nestin*-*Cre;C9orf72*^*fl/fl*^ mice, the number of Lamp1-, 2-, and 3-positive lysosomes is decreased. Together, these data support a model in which C9ORF72 haploinsufficiency inhibits endosomal maturation and consequently induces a decrease in the number of lysosomes and in protein degradation. **c** C9ORF72 patient-derived and C9ORF72 deficient iMNs show hyperexcitability and enhanced cell surface expression of glutamate receptors, which may lead to glutamate toxicity. **d** C9ORF72 loss- and gain-of-function mechanisms may cooperate. Reduced protein degradation as a result of C9ORF72 haploinsufficiency may facilitate the enhanced accumulation of toxic DPRs or other ALS-associated proteins in MNs. **e** Impaired endocytosis of TrkB receptors in C9ORF72 patient-derived MNs negatively affects neuronal survival. This figure was created using Servier Medical Art templates, which are licensed under a Creative Commons Attribution 3.0 Unported License; https://smart.servier.com

The precise mechanism through which hexanucleotide expansions in *C9ORF72* cause motor neuron degeneration is subject of intense study but remains incompletely understood. However, several observations support the idea that surface expression, trafficking, and recycling of cell surface receptors are affected in C9ORF72 ALS/FTD patient cells. For example, in induced motor neurons (iMNs) from C9ORF72 ALS/FTD patients, elevated cell surface levels of the NMDA receptor NR1 and the AMPA receptor GluR1 are found on neurites and dendritic spines compared to control iMNs. Furthermore, glutamate receptors accumulate at post-synaptic densities in these neurons [194]. Elevated levels of glutamate receptors may induce hyperexcitability and cell death due to increased glutamate activation (Fig. 1). In line with this idea, activation of Kv7 potassium channels increases the survival of C9ORF72 patient-derived and C9ORF72-deficient iMNs [194]. Another class of transmembrane receptors affected by *C9ORF72* mutations are Mannose-6-phosphate receptors (M6PRs) [194]. In iMNs from patients with *C9ORF72* mutations, M6PRs cluster and move at slower rates as compared to control [194]. Another study shows that M6PRs localize in the cytosol of C9ORF72 ALS/FTD fibroblasts in contrast to their perinuclear localization in control cells [5]. Given the role of M6Rs in targeting lysosomal enzymes to lysosomes these changes could affect lysosomal degradation (Fig. 1).

Elevated cell surface levels of NMDA and AMPA receptors and defective trafficking of M6PRs in C9ORF72 patient-derived and C9ORF72-deficient iMNs could result from defects in multiple steps of the intracellular trafficking pathway. Interestingly, several studies show that endocytosis and recycling mechanisms are impaired in C9ORF72 ALS/FTD. For example, decreased expression of Vps26, a component of the retromer complex [22], found in C9ORF72 ALS/FTD fibroblasts could lead to abnormal endosomal recycling [5]. Furthermore, knockdown of C9ORF72 in SH-SY5Y cells causes impaired endocytosis of tropomyosin receptor kinase receptor B (TrkB) [47]. In addition, multiple lines of experimental evidence link C9ORF72 to Rab-GTPases. Rab-GTPases control different steps of the intracellular trafficking pathways including vesicle formation, movement and membrane fusion (for review, see [162, 199]).

Rab-GTPases alternate between two conformational states: the activated guanosine tri-phosphate (GTP)-bound state and the guanosine di-phosphate (GDP)-bound inactive state. Exchange of GDP with GTP is catalyzed by Rab guanine-nucleotide exchange factors (GEFs) that act at specific membranes and facilitate GDP release. In contrast, GAPs (GTPase activating proteins) catalyze GTP hydrolysis to GDP [162, 199]. It has been found that C9ORF72 contains a DENN-like domain [100, 196] which acts as an Rab-GEF [77]. In addition, C9ORF72 binds several of the over 60 human Rab-GTPases. To investigate with which Rab-GTPases C9ORF72 can interact, four groups performed interactome experiments. In SH-SY5Y cells, C9ORF72 colocalizes and co-immunoprecipitates (IPs) with Rab1, Rab5, Rab7, and Rab11 [47]. Another study, using C9ORF72 overexpression in COS-7 cells, did not find interactions with the above-listed Rab-GTPases but with a large number of other Rabs (Rab5A, 8A, 10, 13, 15, 18, 19, 27A, 28B, Rab7L1, 38, 40A, and 42) [5]. However, another very recent study in HEK293 cells reported interactions between C9ORF72 and Rab3A, 3B, 3C, and 3D, but not with Rab1A, Rab7A and Rab5A [53]. Finally, a fourth study did not detect any of these interactors nor other proteins involved in autophagy or endocytosis in neuronal cells (N2A cells) overexpressing C9ORF72. Instead, this study reports an enrichment for proteins with mitochondrial functions [15]. One explanation for these discrepancies is that interactome composition is strongly influenced by bait expression levels, experimental set-up, or cell type. Nevertheless, these observations support a strong link between C9ORF72 and Rab-GTPases.

In iMNs, C9ORF72 strongly colocalizes with Rab5-positive endosomes (Fig. 1) and rarely with Lamp1-positive late endosomes/lysosomes [194]. Density gradients reveal that C9ORF72 co-segregates with light fractions (positive for EEA1) but not with heavy fractions (positive for Lamp1) [194], suggesting that C9ORF72 acts primarily on early endosomes. Interestingly, in C9ORF72 ALS/FTD iMNs from patients and spinal motor neurons in *Nestin*-*Cre;C9orf72* ^*fl/fl*^ mice, the number of Lamp1-, 2-, and 3-positive vesicles is decreased, as compared to control [194]. This implicates C9ORF72 in the control of protein degradation. For protein degradation through the endosomal pathway (for autophagy, see Sect. 3), early endosomes need to mature. The transformation from early to late endosomes requires a so-called Rab5/Rab7 conversion. Conversion from Rab5 to Rab7 on early endosomes involves interactions between activated Rab-GTPases and Vsp26, Vsp29, and Vsp35 [105]. Therefore, disturbed Rab function and decreased Vsp26 levels [5] due to *C9ORF72* haploinsufficiency may converge during this step of endosomal maturation. In all, these observations support a model in which reduced C9ORF72 expression maintains early endosomes in an inactive state, preventing maturation and eventually protein degradation. Reduced degradation may have several consequences. As outlined above, it could contribute to hyperexcitability and glutamate toxicity. Furthermore, changes in TrkB signalling may promote MN degeneration, as these receptors normally regulate cell survival [193]. TrkB has been shown to signal from Rab5- and Rab7-positive endosomes [67]. Decreased endocytosis of TrkB receptors in neurons with decreased C9ORF72 expression could affect signalling cascades regulating neuronal survival (Fig. 1). Finally, reduced protein degradation may contribute to the accumulation of proteins or DPRs in aggregates, a hallmark of ALS pathology [15] (Fig. 1).

Disrupted receptor trafficking in ALS has also been linked to TDP-43 (TAR-DNA-binding protein of 43 kDa). TDP-43 binds RNA and modulates multiple RNA processes including RNA synthesis, splicing, stability, and transport [103]. It is likely to function in multi-protein/RNA complexes [51, 102, 153], is involved in regulation and biogenesis of miRNAs [21, 82], and binds DNA which leads to repression of gene transcription [1]. In normal brain, TDP-43 localizes to the cell nucleus [124]. However, SALS cases and most FALS cases (together over 95% of ALS patients) display cytoplasmic TDP-43 inclusions accompanied by nuclear depletion of the protein in affected cells [107, 161]. In addition, some FALS patients have *TDP*-*43*-specific gene mutations that affect TDP-43 localization and function [151]. Most of the work linking TDP-43 to intracellular receptor trafficking derives from experiments that use knockdown or overexpression of TDP-43. TDP-43 knockdown leads to reduced ErbB4 and EGFR1 cell surface expression because of delayed surface recovery following receptor activation [150], which suggests defects in receptor recycling. EGF–EGFR signalling promotes survival, maturation, and outgrowth of neurons [25, 90] and, therefore, decreased EGFR1 cell surface expression, as a consequence of TDP-43 knockdown, is likely to affect neuronal survival or axonal innervation.

TDP-43 depletion in *Drosophila* affects BMP receptors. BMP signalling occurs on the early endosomes from where BMP receptors enter either the recycling pathway to the cell surface or are targeted for endo-lysosomal degradation [36]. Deshpande et al. show that synaptic growth is facilitated through the phosphorylation of MAD downstream of BMP receptors. MAD phosphorylation is significantly decreased in *Drosophila* motor neurons following overexpression or depletion of TDP-43 [35]. On basis of these observations, the authors suggest that reduced signalling of BMP receptors caused by non-equilibrated TDP-43 levels induces receptor missorting towards the early/recycling endosomes ultimately affecting neuronal and synaptic growth [35].

Alsin-2 is another gene that has been linked to defective endosomal trafficking in ALS. Alsin-2 (**ALS2**) is a 185 kDa protein which contains an ATS1/RCC1-like domain, a RhoGEF domain, and a vacuolar protein sorting 9 (VPS9) domain. Alsin-2 functions as a guanine-nucleotide exchange factor (GEF) for Rab5 and localizes with Rab5 on the early endosomal compartments. At least 12 different mutations in ALS2 have been reported in juvenile ALS and primary lateral sclerosis (PLS). These mutations have been described as frameshift, missense, or nonsense mutations [26]. Of these, two mutations found in the RCC-1 domain result in frame shift mutations that cause juvenile ALS (*ALS2*) [92, 132, 192]. Co-expression of Rab5 and a truncated form of Alsin-2 (containing MORN motifs and the VPS9 domain) prevents endosomal fusion. To establish the role of ALS2, effects on endosomal trafficking were studied in an *Alsin*-*2*^−*/*−^ mouse model. Hippocampal neurons of *Alsin*-*2*^−*/*−^ mice show accumulation of Rab5-positive endosomes, decreased Rab5 mobility, and increased colocalization of Rab5 with Lamp1. In addition, *Alsin*-*2*^−*/*−^ hippocampal neurons show faster degradation of AMPA receptors following stimulation [94]. These experiments suggest that Rab5 endosomes remain in their GDP-bound state and, consequently, interact to a lesser extent with downstream effectors. Inactive downstream effectors, such as the sorting machinery or motor proteins, could lead to decreased transport, decreased sorting (either into the recycling or the degradative pathway), and affect receptor activation due to decreased recycling.

Some ALS mutations are thought to affect endosomes at the level of lysosomal degradation. ALS-associated mutations in charged multivesicular body protein 2B (***CHMP2B***) and spastic paraplegia-11 (***SPG11***) have been reported to deregulate endosomal trafficking towards degradation. Loss-of-function of SPG11 affects endo-lysosomal homeostasis, anterograde trafficking, and lysosomal turnover [34]. CHMP2B is a subunit of the endosomal-sorting complex required for transport-III (ESCRT-III) which is required for the formation and fission of intra-luminal vesicles in the late endosomes/multivesicular bodies. Proteins within intra-luminal vesicles are then delivered to lysosomes for degradation via endo-lysosomal fusion. Mutations in *CHMP2B* lead to lysosomal storage pathology and a decrease in neuronal endo-lysosomal motility. Interestingly, this trafficking defect could be rescued by knockdown of the FTD risk factor TMEM106B [43]. Mutations of *CHMP2B* and *SPG11* could increase susceptibility to neuronal death by deregulation of protein degradation.

Exome sequencing revealed that mutations in valosin-containing protein **(VCP/p79** or **ALS14)** account for 1–2% of FALS [79]. VCP/p97 regulates endo-lysosomal sorting of ubiquitinated cargos such as caveolin-1 and loss of VCP/p97 accelerates the accumulation of autophagosomes. [133]. Therefore, VCP/p97 mutations may affect maturation and degradation of autophagosomes and endo-lysosomes through impaired fusion with lysosomes [79, 133].

Another protein that may link ALS and processes such as endosomal trafficking and protein degradation is Fig4. Fig4 is a member of the SAC phosphatase family which removes the 5-phosphate from PI(3,5)P2 to form PI(3)P [24, 42, 48, 146, 178]. Phosphatidylinositol 3-phosphate (PI(3)P) and its subclasses mediate several cellular functions such as membrane identity, endosomal trafficking, signalling, autophagy, and degradation [109]. The generation of these distinctive pools of PI3-phosphates occurs via three classes of PI3-kinases and different PI(3)Ps can localize to distinct endosomal compartments and thereby define membrane identity [109]. Fig4 forms a complex with two other proteins: VAC14, a scaffolding protein, and FAB1, a kinase that generates PI(3,5)P2 from PI(3)P [78]. Mutations in *FIG4* have been found in patients with ALS [29, 131] and at least 14 rare non-synonymous FIG4 variants were detected in ALS cases in a group of 201 central European ALS patients [131]. ALS variants include two protein-truncation mutations, two mutations in consensus splice sites, and six missense mutations, all suspected to interfere with protein function [29]. However, the contribution of FIG4 variants to ALS needs further genetic confirmation since no deleterious FIG4 variants have been reported in larger cohorts. In addition, some non-penetrant FIG4 variant carriers have been described [131]. Nevertheless, Fig4 is an interesting candidate and its altered expression or function may affect endosomal maturation in ALS. For example, the conversion from PI(3)P to PI(3,5)P2 is crucial for endosomal maturation, since Rab5/Rab7 conversion and the synthesis of PI(3,5)P_2_ from PI(3)P regulate transformation from early to late endosomes. PI(3,5)P2 has been shown to modulate several important functions at late endosomes/lysosomes, and thereby cargo degradation [130, 183]. Future studies should focus on establishing whether ALS-associated *FIG4* variants affect PI(3,5)P2 levels and thereby disrupt the degradative pathway, e.g., by impaired endosomal maturation.

In conclusion, several of the gene defects that have been identified in ALS patients suggest a role for defective endosomal and receptor trafficking in the pathogenesis of this neuromuscular disease. Evidence is strongest for patients carrying hexanucleotide repeat expansions in C9ORF72. C9ORF72 is a Rab-GEF, binds a large number of different Rab-GTPases, and has a role in vesicle transport. C9ORF72 ALS/FTD patient cells show defects in lysosomal degradation and cell surface accumulation of receptors such as glutamate receptors. This may lead to enhanced neuronal excitability and glutamate toxicity. Furthermore, C9ORF72 loss of function is likely to cooperate with gain of function mechanisms, leading, for example, to accelerated DPR accumulation. Manipulation of the expression of TDP-43 and Alsin-2 also induces receptor and endosomal trafficking defects, while it will be interesting to assess whether *FIG4, VCP, CHMP2B,* or *SPG11* variants associated with ALS affect these pathways, as well. Overall, these observations suggest a central role for receptor and endosomal trafficking in the pathogenesis of ALS.

## Autophagy dysregulation

A key pathological hallmark of ALS is the mislocalization of (disease-associated) proteins and the formation of protein aggregates [15]. Defective protein degradation contributes to these pathological events and abnormal autophagy has been linked to ALS. Autophagy is an important protein degradation pathway involved in the clearance of protein aggregates and damaged organelles. It is highly dependent on intracellular transport of vesicles (e.g., lysosomes and autophagosomes) by motor proteins and, therefore, discussed in this review. Three types of autophagy have been described: microautophagy, macroautophagy, and chaperone-mediated autophagy. Macroautophagy is the main pathway used to eradicate damaged organelles and proteins [61] and is discussed below in relation to a few key ALS-associated proteins. For a more extensive discussion on the role of autophagy in ALS, see [45, 139].

Autophagy is initiated by the assembly of an isolation membrane with a cup-like shape. This membrane, the so-called phagophore, is built at the phagophore-assembly site (PAS) which is a nucleated site reported to be on ER, ER–mitochondria, ER–plasma membrane, as well as the plasma membrane, Golgi complex, and recycling endosomes (for review, see [38, 61]). The molecular details of phagophore nucleation are incompletely understood, but involve recruitment of ATG proteins to the PAS. There, ATG proteins interact with other proteins, and according to these interactions, ATGs are grouped into five complexes (for review, see [38, 61]). One of these complexes, the class 3 PI3-Kinase complex, is targeted by superoxide dismutase 1 **(SOD1)**. SOD1 is a homo-dimeric metalloprotein that dismutates free superoxide radicals that cause oxidative stress. Over 180 mutations have been found in *SOD1*. VPS34 converts PI into PI(3)P and interacts with Beclin1, p115, ATG14, and PI3-Kinase in one complex. Mutant SOD1 impedes the vesicle nucleation step of autophagy through abnormal interaction with Beclin-1 and, consequently, destabilization of the Beclin-1–Bcl-x_L_ complex [98].

Fused in sarcoma (**FUS**) is a DNA/RNA-binding protein with functional homology to TDP-43 [96]. It associates with the transcription machinery and influences transcription initiation and promoter selection [93]. Many *FUS* mutations in ALS affect its nuclear localization signal (NLS) and mutant *FUS* is thought to act through both gain- (protein aggregation) and loss-of-function (nuclear depletion) mechanisms [95, 107]. FUS is a major component of cytoplasmic stress granules and these FUS-containing stress granules colocalize with autophagosomes. When mutant FUS is overexpressed in primary neurons, autophagy is decreased, while, simultaneously, the number of FUS-positive stress granules is increased [147]. Another study shows that overexpression of *FUS*^P525L^ and *FUS*^R522G^ impairs autophagy in neuronal cell lines and primary cortical neurons. Here, mutant FUS expression results in the formation of fewer omegasomes, which are precursors to autophagososomes. In addition, these precursors recruit less ATG9 and lipidated LC3-II, required for autophagy initiation and elongation. Therefore, mutant FUS appears to inhibit autophagy by interfering with early autophagosome formation.

Several lines of evidence link **TDP-43** to autophagy. TDP-43 aggregates colocalize with autophagy markers such as LC3 and p62/SQSTM1 (Sequestosome 1 (SQSTM1, also known as ubiquitin-binding protein p62) [70]. Furthermore, VCP and optineurin (OPTN), which colocalize with TDP-43, p62/SQSTM1, and ubiquitin, colocalize in spinal motor neurons of sporadic ALS patients [11]. Furthermore, elevated levels of LC3 have been found in skin biopsies of patients carrying the TDP-43^A315T^ mutation, suggesting that ALS-associated TDP-43 mutations may enhance autophagy [180]. In all, these data link SOD1, FUS, and TDP-43 mutations to autophagy dysregulation, suggesting that part of the pathogenic effects of these mutations may derive from their ability to affect protein or organelle removal.

In **C9ORF72** ALS/FTD patients, p62, a protein targeting cargo for autophagy, accumulates in the cerebellum, hippocampus, and neocortex, suggesting impaired autophagy [3, 30, 108]. P62 interacts with C9ORF72 [152], and increased levels of p62 are detected in C9ORF72 patient-derived fibroblasts [5]. Furthermore, following C9ORF72 knockdown in neurons, autophagy is impaired and both p62 and TDP-43 accumulate in aggregates [152]. This accumulation may be explained by defective Rab signalling as several of the Rabs that are involved in the formation of autophagosomes bind the Rab-GEF C9ORF72 (such as Rab1A, 8A, and 39B) [31, 182]. In addition, C9ORF72 forms a complex with SMCR8 and WDR41 [152], which act as GDP/GTP exchange factors for Rab8A and Rab39B. Another Rab-GTPase linked to C9ORF72-mediated autophagosome formation is Rab1A [5]. C9ORF72 interacts with Rab1A and the ULK1 complex to regulate initiation of autophagy [182]. Two studies, using HeLa and SH-SY5Y cells, report that depletion of C9ORF72 reduces the formation of LC3-positive autophagosomes [47, 182], which are double-membrane vesicles that deliver cargo to lysosomes for degradation, while overexpression increases autophagy [182]. Other work reports increased levels of p62 and LC3 in Western Blots from C9ORF72 patient fibroblasts. Similarly, analysis of mouse embryonic fibroblasts (MEFs) and neuronal precursors from *C9ORF72*^−/−^ stem cells shows an increase in LC3 [175]. This study suggests that higher levels of LC3 would indicate higher levels of autophagy. However, transmission electron microscopy of iMNs from C9ORF72 ALS patients reveals swollen and, therefore, likely non-functional, autophagosomes. This could indicate disrupted degradation of autophagosomes and decreased autophagy [5]. Another study reports increased autophagy flux caused by an increase in transcription factor EB (TFEB), a master regulator of lysosome biogenesis [154]. Consistent with increased autophagy flux, this study detects a decrease in p62 levels in brain tissue from *C9ORF72*^−*/*−^ mice and in *C9ORF72*^−*/*−^ MEFs [175]. The apparent discrepancies between these different studies may result from the use of different cell types (i.e., HeLa cells, SH-SY5Y cells, MEFs, neuronal precursors, and iMNs) or from the use of different experimental approaches to decrease or deplete C9ORF72 (ranging from siRNA-mediated knockdown to depletion of C9ORF72 in KO mice and patient-derived C9ORF72 iMNs with lower *C9ORF72* expression, RNA foci and DPRs). Nevertheless, these data link disrupted autophagy to C9ORF72 hexanucleotide repeat expansions and C9ORF72 loss of function. Reduced protein degradation may contribute to the accumulation of proteins or DPRs in aggregates, a hallmark of ALS pathology [15] (Fig. 1). Therefore, in C9ORF72 ALS/FTD, loss and gain of function phenotypes (e.g., glutamate toxicity and impaired clearance of toxic DPRs) may cooperate to induce MN degeneration, as has been suggested on basis of work in C9ORF72 patient-derived deficient iMNs [194]. Here, the authors suggest that C9ORF72 haploinsufficiency may trigger defects in lysosomal biogenesis that impair the clearance of DPRs, generated from C9ORF72 repeat expansions, thereby exacerbating the toxic effects of these polypeptides.

Mutations in ubiquilin-2 (**UBQLN2**) cause X-linked juvenile and adult-onset ALS and ALS/dementia [185]. ALS-associated mutations in UBQLN2 cause a failure in the delivery of cargo to the proteasome leading to defective protein degradation and cell toxicity [27].

Mitophagy is removal of damaged mitochondria through autophagy. This removal is regulated by the PINK1-PARKIN pathway which poly-ubiquitinates damaged mitochondria to promote mitophagy. This ubiquitination recruits Tank-binding kinase 1 (**TBK1)** which phosphorylates OPTN and p62, both of which serve as autophagy adaptors for ubiquitinated proteins targeting them to the phagophore [129, 134, 188]. Mutations in ***OPTN*** (aka FIP-2) are associated with normal tension glaucoma and ALS. OPTN binds ubiquitin and functions as an autophagy receptor [184]. The addition of poly-ubiquitin chains by PINK-PARKIN, as well as phosphorylation by TBK1, promotes the rapid recruitment of OPTN, nuclear dot protein 52 kDa (NDP52), SQSTM1/p62, and Tax1-binding protein 1 (TAX1BP1) to damaged mitochondria, and this recruitment is blocked by inhibition or deletion of TBK1. TBK1 loss of function has been reported to cause FTD/ALS [60, 129], possibly by affecting autophagosome formation due to reduced phosphorylation and recruitment of OPTN and p62. Following phosphorylation and association with damaged mitochondria, OPTN recruits LC3 via its LC3-interacting region (LIR) domain, resulting in autophagosome formation around mitochondria [69, 97, 188]. At a later stage in autophagy, OPTN binds the myosin VI motor protein, facilitating autophagosome maturation and fusion to lysosomes (see below within the C9ORF72/SMCR8/WDR41 pathway) [174].

In addition to its role in autophagy, TBK1 is an NF-kB effector by phosphorylating the NF-kB inhibitors alpha/NFKBIA, IKBKB, or RELA to translocate NF-kB to the nucleus. NF-kB activation can occur during signalosome assembly downstream of OPTN [200]. The NF-kB protein complex is crucial for the regulation neuronal survival and acts downstream of TrkB activation [8, 23] linking TBK1 to survival signalling downstream TrkB receptors.

In conclusion, the identification of ALS-associated genetic defects in *SOD1*, *FUS*, *TDP*-*43*, *C9ORF72*, *TBK1,* and *OPTN,* and the abnormalities in protein homeostasis and autophagosomes associated with these defects support an important role for dysregulated autophagy in ALS pathogenesis. Most studies show reduced autophagy due to hexanucleotide expansions or C9ORF72 loss of function, but other data hint at enhanced autophagy. Therefore, further work is needed to dissect the precise autophagy defects associated with C9ORF72 pathology. Similarly, while mutant SOD1, FUS, and TDP-43 affect autophagy and TBK1 and OPTN play a role in autophagy, the precise pathological effects of ALS-associated mutations in these genes remain incompletely understood. However, it is clear that altered autophagy plays a key role in ALS pathogenesis and contributes to motor neuron degeneration by inducing a failure to remove toxic proteins and damaged organelles, and allowing accumulation of proteins in aggregates.

## Altered transport to and from ER and Golgi in ALS

In Sects. 2 and 3, a role for C9ORF72 in endosomal transport and autophagy was discussed. However, **C9ORF72** has also been linked to disrupted ER–Golgi transport in ALS. Once synthesized, proteins targeted for the secretory pathway enter the endoplasmic reticulum (ER). There, proteins are folded by ER-resident enzymes and chaperones [17]. Correctly folded proteins leave the ER at specialized sites called ER exit sites (ERES) via coat protein complex II (COPII)-coated vesicles and form tubular-like structures known as the ER–Golgi intermediate compartment (ERGIC) and subsequently fuse with the Golgi [6, 145]. COPII-coat-mediated transport between the ER and Golgi is facilitated by Rab1 and Rab2, while trafficking from ERGIC back to the ER is regulated by Rab2 in a COPI-dependent fashion or by Rab6 in a COP-independent manner [18, 68]. In the ERGIC, additional sorting takes place where proteins are either transported further towards the Golgi or are recycled back to the ER [64, 111]. Several lines of experimental evidence suggest that dysregulation of C9ORF72 may impact these different transport routes. First, C9ORF72 associates with Rab1 and may modulate COPII-dependent ER–Golgi transport [47]. Furthermore, C9ORF72 knockdown impairs endocytic trafficking from the plasma membrane to Golgi [5]. This is in line with the interaction of C9ORF72 with Rab11 endosomes, which mediate recycling from the plasma membrane through the trans-Golgi network (TGN) [47]. Finally, C9ORF72 is GEF for Rab8 [152], which regulates vesicular traffic between the TGN and the basolateral plasma membrane [73]. Another ALS-associated protein known to interact with Rab8 is **OPTN** [173]. It has been hypothesized that ALS mutations in *OPTN* affect trafficking of Rab8-positive endosomes [122]. Together, these data link ALS-associated proteins via Rab-GTPases to intracellular trafficking to and from ER/Golgi. The precise functional effect of ALS mutants on this trafficking and the underlying molecular mechanisms require further study.

Members of the vesicle-associated membrane protein-associated protein **(VAP)** family, such as VAPA and VAPB, are present in the ER and regulate ER and Golgi transport to maintain Golgi complex identity and ER morphology, as well as lipid transfer [99, 137, 160, 171]. In addition, VAPB interacts with ER–Golgi recycling proteins and modulates the delivery of membrane to dendrites [91]. A dominant missense mutation, P56S, in the MSP domain of VAPB causes FALS8 [127]. VAPB^P56S^ accumulates in inclusions containing disrupted ER [135, 171]. Furthermore, it recruits wild‐type VAPA/B into such inclusions. It has, therefore, been proposed that VAPB^P56S^ has a dominant‐negative effect on VAP function [171]. Interestingly, VAP protein levels are reduced in SALS patients, SOD1 mutant mice, and ALS patient‐derived MNs [4, 113, 171]. YIF1A, a transmembrane protein that plays an important role in secretion [12, 195], binds to VAPB and the ALS-mutant VAPB^P56S^ [91]. YIF1A cycles between the ER and Golgi and is mainly localized to the ERGIC. However, following expression of the ALS-mutant VAPB^P56S^, YIF1A fails to localize to the ERGIC causing the disruption of this structure. This event has been suggested to contribute to motor neuron degeneration [91].

Mutations in **SOD1** affect trafficking at the Golgi in several ways. For example, the ALS-associated mutations *SOD1*^*A4V*^, *SOD1*^*G85R*^, and *SOD1*^*G93A*^ disrupt the secretory pathway. In one study, BDNF was used as a marker of ER–Golgi protein secretion in the presence of SOD1 mutant protein and BDNF levels were significantly higher in conditioned medium of untransfected cells, cells expressing SOD1^WT^ or EGFP controls as compared to medium from SOD1^A4V^, SOD1^G85R^, and SOD1^G93A^ transfected cells. In contrast, intracellular levels of BDNF in SOD1^A4V^, SOD1^G85R^, and SOD1^G93A^ transfected cells were higher as compared to controls, hinting at defects in secretion [9]. In addition to defects in secretion, the SOD1^A4V^ mutation triggers ER stress causing inclusions and apoptosis, while overexpression of COPII rescues the above-mentioned *SOD1*-mutant phenotypes. This suggests that misfolded SOD1 interferes with ER-to-Golgi trafficking via COPII vesicles. Overall, these defects in the secretory pathway and the resulting accumulation of secretory proteins could explain how SOD1-induced ER stress leads to apoptosis [9].

In addition to the secretory pathway, SOD1 mutations inhibit transport from ERGIC to Golgi, but not from ER to ERGIC [159]. ER-to-ERGIC transport is affected by mutant **TDP-43** and mutant **FUS**. Both affect Rab1-dependent trafficking of COPII vesicles, which disrupts transport from ER to ERGIC. Interestingly, overexpression of Rab1 rescues this defect [104, 159]. TDP-43 is localized at the cytoplasmic face of the ER membrane, while FUS localizes within the ER, inhibiting transport at two different sites [159]. Even though the exact mechanism through which Rab1 trafficking is affected remains elusive, disrupted vesicle trafficking from Golgi has been reported to cause Golgi fragmentation, a hallmark of many neurodegenerative diseases, including ALS [55, 56, 62]. It has been shown that depletion of Golgi-associated Rabs causes destabilization of the Golgi [57]. Interestingly, Golgi-associated vesicular trafficking is inhibited in cells expressing different ALS-mutant proteins (SOD1, FUS, TDP-43, and OPTN [10, 39, 159, 165]). Defective ER–Golgi transport precedes all other cellular pathologies in addition to fragmentation, including ER stress, protein aggregation, inclusion formation, and apoptosis [9]. Overall, these data suggest that altered ER–Golgi transport may be one of the first disease mechanisms through which ALS-mutant proteins affect motor neuron function and survival.

Apart from protein folding and quality control, the ER forms structural connections with mitochondria, which facilitate a number of cellular functions such as energy metabolism, Ca^2+^ homeostasis and lipid metabolism. Interestingly, mitochondria-ER contact sites occur on acetylated microtubules [54] (see Sect. 5). The disruption of ER–mitochondria associations has been linked to Alzheimer’s disease [7] and ALS. **VAPB** proteins localize to the ER and interact with mitochondrial protein tyrosine phosphatase-interacting protein-51 (PTPIP51) to regulate ER–mitochondria interactions. TDP-43, both wild-type and ALS mutants TDP-43^M337V^, TDP-43^Q331K^, TDP-43^A382T^, or TDP-43^G348C^, perturb ER–mitochondria interactions by disrupting VAPB–PTPIP51 binding [164]. In addition to TDP-43, FUS also affects ER–mitochondria interacting sites. FUS activates GSK3-β which disrupts VAPB–PTPIP51 and ER–mitochondria interactions [163]. It is, therefore, tempting to speculate that mutations in VAPB, TDP-43, and FUS may contribute to ALS pathogenesis by interfering with energy metabolism at the level of ER–mitochondria interactions.

In conclusion, trafficking pathways from and to the ER and Golgi complexes, and between ER and Golgi, are a major target in ALS. In part, these defects involve Rab-GTPases and their effectors. The precise molecular details of how these defects result in motor neuron degeneration remain largely unknown. However, defective trafficking from Golgi can lead to Golgi fragmentation, a major hallmark of ALS and other neurodegenerative diseases. Interestingly, some of the reported defects (ER-to-ERGIC) can be rescued by Rab1 overexpression, highlighting Rab-GTPases as potential therapeutic targets. Moreover, perturbations in ER–mitochondria contacts as a consequence of VAPB, TDP-43, or FUS mutations could affect energy metabolism, Ca^2+^ homeostasis, and lipid metabolism.

## Disrupted axonal transport in ALS

**Fig. 2:**
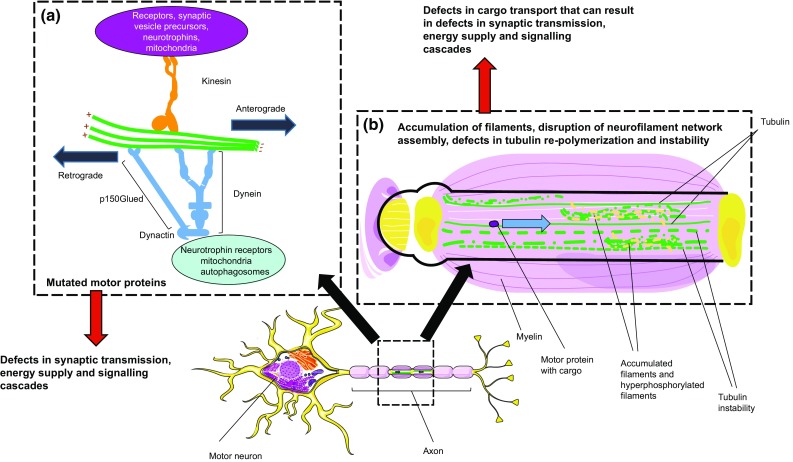
ALS-associated mutations disrupt axonal transport by affecting motor and cytoskeletal proteins. **a** ALS-associated mutations in the C-terminal part of kinesin-5A (KIF-5A), a member of the kinesin family, cause ALS. These mutations are thought to affect cargo binding. Other ALS-associated mutations, such as in *SOD1* or *FUS*, affect kinesins indirectly, e.g., by altering expression levels or phosphorylation state. Mutations in p150^Glued^, a subunit of the dynein/dynactin complex, have been implicated in ALS and affect binding of p150^Glued^ to microtubules. Defective motor proteins have been firmly linked to ALS pathogenesis and may affect motor neuron physiology by dysregulating the transport of essential cargo such as mitochondria, autophagosomes, growth factors, and signalling cues. **b** Accumulation of cytoskeletal proteins such as neurofilaments is a pathological hallmark of ALS. ALS-associated mutations in SOD1, FUS, TDP43, and C9ORF72, but also in cytoskeletal proteins, such as neurofilament heavy chain (NFH), peripherin (PRPH), and tubulin beta-4A (TUB4A), cause accumulation of cytoskeletal proteins, disruption of neurofilament network assembly, decreased re-polymerization, and reduced actin-binding abilities. Eventually, these defects will disrupt cargo transport and, consequently, affect synaptic transmission, energy supply, and signalling cascades. This figure was created using Servier Medical Art templates, which are licensed under a Creative Commons Attribution 3.0 Unported License; https://smart.servier.com

Axonal transport of cargo such as mitochondria, signalling endosomes, or proteins between the cell body and distant cellular sites, e.g., synapses, is essential for neuronal function and survival (Fig. 2). Defects in axonal transport are linked to ALS and mutations in different proteins that form the axonal transport machinery have been reported in ALS patients. Changes in axonal transport are one of the first pathological hallmarks of ALS and may be an early and key pathogenic event. Two main classes of axonal transport are distinguished, i.e., slow and fast axonal transport, both of which appear to be affected in ALS. These types of transport differ with respect to the speed by which cargo is moved, but both are mediated by the same molecular machinery. Axonal cargo is moved in antero- and retrograde directions by motor proteins, such as kinesins and dynein, along microtubule polymers. Other proteins linked to axonal transport have been implicated in ALS, but, here, we focus on affected motor proteins and the cytoskeleton for which genetic and pathological evidence is strongest. For a more extensive overview and discussion on axonal transport defects, specific cargos, and other transport proteins in ALS, we refer to the other recent reviews [83, 179].

The human kinesin superfamily of molecular motor proteins contains 45 members which mediate both slow and fast anterograde axonal transport. Strongest evidence for the involvement of kinesins in ALS pathogenesis is provided by three studies that reveal mutations in kinesin-5A **(KIF5A)** that cause ALS [20, 198, 126] (Fig. 1). This work identifies mutations in the KIF5A C-terminus which binds cargo-adaptor proteins [123, 140]. These mutations are, therefore, predicted to cause disruption of cargo-binding inducing loss-of-function phenotypes [20]. This hypothesis is in line with the observation that *Kif5a*^−/−^ mice show neurodegeneration and paralysis [191]. Intriguingly, mutations in the kinesin motor domain and coiled-coil domain within the N-terminal part of KIF5A have been reported in Hereditary spastic paraplegia (HSP) and Charcot–Marie–Tooth (CMT) patients [65, 80]. This indicates that KIF5A may be a central target in motor neuron disease.

Kinesins are likely to also be affected indirectly in ALS, for example by mutations in FUS and SOD1. Mutations in **FUS** dysregulate *KIF5C*, *KIF1B*, and *KIF3A* mRNA expression [72] and could thereby modulate cargo transport. ALS-associated SOD1 and FUS mutants also impair fast axonal transport [59, 117, 149]. Furthermore, disruption of anterograde transport by misfolded human **SOD1**^H46R^ protein in isolated squid axoplasm involves p38 MAP Kinase (p38 MAPK) activation and kinesin-1 phosphorylation [16]. p38 MAPK phosphorylates kinesin-1, thereby inhibiting its translocation along microtubules [117]. Inhibition of p38 MAPK protects mutant *SOD1*-expressing motor neurons [37]. How p38 MAPK inhibition acts neuroprotective is unknown but may involve effects on kinesin activity.

Retrograde axonal transport is facilitated by the motor protein dynein 1 [71]. Dynein 1 is composed of two homodimerized dynein heavy chain and multiple dynein intermediate and light chains [87]. The function of this complex is regulated by adapter complexes including dynactin. The dynactin complex contains p150^Glued^ together with other proteins. Mutations in *DCTN1*, which encodes **p150**^**Glued**^, are reported in both SALS and FALS patients [120] (Fig. 1). These ALS-causing mutations impede binding of p150^Glued^ to microtubules, resulting in dysfunctional dynein/dynactin-mediated transport of cargos such as autophagosomes [75, 101]. As described earlier for kinesins, mutations in p150^Glued^ or other components of the dynein/dynactin complex have been linked to various neurological disorders, several of which show motor neuron deficits [83, 179]. Motor neurons from *SOD1G93A* transgenic mice display defective dynein-mediated retrograde transport, both in vitro and in vivo, linking *SOD1* mutations to dynein-mediated transport [14, 84]. Thus, defects in motor proteins are firmly linked to ALS pathogenesis and are predicted to affect motor neuron physiology through disruption of the transport of cargos such as mitochondria, growth factors, and RNA, thereby affecting energy metabolism, survival, and local translation.

Proper axonal transport not only relies on motor proteins but also on cytoskeletal components such as neurofilaments and microtubules. The accumulation of neurofilaments is a pathological hallmark of both FALS and SALS [121, 186] (Fig. 1). The deletion of neurofilament in *SOD1* transgenic mice delays disease onset and reduces progression of ALS pathology. This indicates that neurofilaments contribute to motor neuron toxicity [186]. Accumulation of neurofilaments or disruption of neurofilament growth could lead to stalling of cargo transport in neurons. Interestingly, the accumulation of neurofilaments in ALS may be caused by defects in kinesins and dynein, since neurofilament oligomers are transported by these motor proteins [138, 166].

Several different mutations in cytoskeletal proteins have been reported in ALS. These have been proposed to induce motor neurodegeneration by disturbing the cytoskeleton and disrupting cargo transport. For example, motor neurons of ALS patients with mutations in *neurofilament heavy chain***(*NFH*)** display accumulations of phosphorylated neurofilament proteins that could disrupt axonal transport [115, 121]. Mutations in *peripherin***(*PRPH*)** may act through a similar pathogenic mechanism. Peripherin is a neuronal intermediate filament protein and an ALS-causing frameshift mutation in *PRPH* disrupts neurofilament network assembly [63]. Furthermore, periperin localizes to Bunina bodies, which are small eosinophilic intra-neuronal inclusions in the remaining lower motor neurons [114], which could affect cargo transport. Furthermore, ALS-causing mutations in *tubulin β*-*4A***(*TUB4A*)** destabilize the microtubule network and reduce re-polymerization [158], therefore possibly affecting transport of cargo and microtubule assembly. Mutant SOD1 also affects microtubule dynamics, in part by interacting with tubulin or microtubule-associated proteins (for review [83, 179]).

Studies showing dysregulation or the therapeutic potential of HDAC6 (a class IIb histone deacetylase involved in microtubule stability [32]) in ALS further highlight the role of microtubules in ALS. ALS-associated mutations in ***FUS*** and ***TDP*-*43*** regulate HDAC6 expression [49, 86, 112]. Acetylation of α-tubulin is important for the binding of molecular motor proteins to microtubules [141, 148] and deletion of *HDAC6* significantly slows disease progression and prolongs survival in mutant *SOD1*^*G93A*^ mice [167]. These data identify HDAC6 inhibition as a potential therapeutic strategy in ALS. This is supported by the observation that HDAC6 inhibition reverses axonal transport defects in iPSC-generated motor neurons derived FUS-ALS patients [66].

In addition to the microtubule cytoskeleton, proteins such as profilin and cofilin have been implicated in ALS and indicate a role for the actin cytoskeleton. Profilin **(*PFN1*)** promotes nucleotide exchange on actin converting monomeric ADP–actin to ATP–actin [116]. PFN1–ATP–actin complexes bind to the fast-growing end of actin filaments regulating filament growth [172]. Patients with mutations in *PFN1* display atrophy of the limbs and it has been suggested that *PFN1* contributes to ALS pathogenesis by altering actin dynamics resulting in axon outgrowth inhibition [189]. This is consistent with the finding that the ALS-associated PFN1 mutants *C71G* and *M114T* display reduced actin binding and inhibit axon outgrowth of embryonic motor neurons [189]. In addition, *PFN1* mutants cause the formation of ubiquitinated, insoluble aggregates that colocalize with TDP-43 [189]. C9ORF72 interacts with cofilin **(CFL1)**, a key regulator of actin dynamics [19]. Knockdown of **C9ORF72** expression reduces axonal growth and actin dynamics [157]. Reduced C9ORF72 levels observed in ALS could affect actin dynamics and thereby, for example, cargo trafficking along the actin cytoskeleton at axonal branches or synapses [13].

In summary, retrograde and anterograde axonal transport is crucial for the distribution of cargo in motor neurons. ALS-associated defects in motor proteins and the cytoskeleton, that both are required for cargo transport, are predicted to cause various molecular and cellular perturbations, e.g., in receptor signalling, synaptic function, gene regulation, energy metabolism, or lysosomal degradation that could lead to motor neuron degeneration. However, our understanding of how defective motor and cytoskeletal proteins cause ALS and specifically affect MNs is rather incomplete. Further studies are needed to fill this void. Genetic and experimental evidence for a role for disrupted axonal transport in ALS pathogenesis is strong and has provided several starting points for the development of therapeutic strategies (for an overview see [83, 179]). For example, inhibition of p38 MAPK protects motor neurons from degeneration and HDAC6 depletion has positive effects on ALS disease progression and survival. However, further work is needed to implement these and other strategies. For examples, HDAC6 not only regulates axonal transport but is also known to play a crucial role in the clearance of protein aggregates by autophagy [81]. Therefore, strategies need to be developed that specifically affect the transport-mediated effects HDAC6.

## Nucleocytoplasmic transport

A major pathological hallmark of ALS is the nuclear depletion and cytoplasmic accumulation of **TDP-43**, which is observed in over 95% of ALS patients [107, 161]. Consistent with this pattern of protein re-distribution, impaired nucleocytoplasmic transport has emerged as a disease mechanism in ALS and other neurodegenerative diseases (for review, see [46, 85]). Several lines of experimental evidence have recently implicated defects in nucleocytoplasmic transport in ALS. These include the presence of mutations in the nuclear localization signals (NLS) of proteins such as FUS and hnRNPA1 [40, 96, 106]. **FUS** causes toxicity in part through the formation of abnormal aggregates in the nucleus and cytoplasm of affected neurons and glial cells in ALS patients with *FUS* mutations [95, 107]. Most of the reported *FUS* mutations in ALS are missense mutations affecting its C-terminal NLS [96]. Interestingly, the ALS-associated protein aggregates that form as a result of impaired nucleocytoplasmic trafficking may themselves also interfere with nucleocytoplasmic transport of protein and RNA [187]. Finally, repeat expanded **C9ORF72** has been proposed to affect trafficking between the nucleus and cytoplasm in different ways. As discussed before, disease-associated repeat expansions in C9ORF72 induce motor neuron degeneration and ALS in part through toxic gain of function mechanisms. These include the accumulation of mutant transcripts and DPRs. Expanded repeat-containing *C9ORF72* transcripts accumulate in affected motor neurons, and other cells in the brain and spinal cord. Several studies suggest that these stable hexanucleotide repeat-containing *C9ORF72* RNA species sequester RNA-binding proteins and nuclear pore complex (NPC) components (e.g., RanGAP1). This disturbs the function and nucleocytoplasmic trafficking of these and other proteins [197]. Mislocalization and accumulation of NPC proteins have also been observed in ALS cases linked to other mutations, such as ***SOD1*** [88, 155]. Another mechanism by which C9ORF72 repeat expansion affects nucleocytoplasmic transport is the generation of DPRs. DPRs have been proposed to block the central channel of the nuclear pore [156]. However, how exactly RNA accumulation or DPRs disrupts nucleocytoplasmic transport remains incompletely understood (for review, see [52, 85]).

Central to nucleocytoplasmic transport is the NPC, a large protein complex that spans the membranes of the nuclear envelope and that is composed of about 30 different nucleophorins. A direct link between the NPC and **C9ORF72** is provided by work identifying components of the NPC (nucleophorins and nuclear transport receptors) as genetic modifiers of C9ORF72-related neurodegeneration and the binding of these components to DPRs (for review, see [85]). Reduced expression or mislocalization of components of the NPC and nuclear import factors, i.e., importins, in ALS brain and spinal cord tissue [88, 125, 128, 169] or caused by TDP-43 aggregation in vitro [28] further implicates transport through the NPC in ALS pathology. Interestingly, mutations in the endosomal-sorting complexes required for transport (ESCRT) III subunit CHMP2B are causative for ALS [43]. Genetic ablation of ESCRT-III in yeast leads to clustering of defective NPCs due to mis-assembly [181]. It will, therefore, be interesting to assess whether CHMP2B mutations lead to defects in NPC assembly surveillance and thereby motor neuron degeneration.

In conclusion, defects in nucleocytoplasmic transport are increasingly recognized as a key event in the pathogenesis of ALS. Evidence for changes in nucleocytoplasmic trafficking are particularly strong in C9ORF72 ALS/FTD. These include mislocalization of proteins forming or associating with the NPC and clogging of the NPC by DPRs. Despite this recent progress, our understanding of how defective nucleocytoplasmic transport leads to motor neuron degeneration and ALS remains rather incomplete. Furthermore, the relative importance of this proposed disease mechanism in comparison to the other ALS disease mechanisms remains to be established. Fundamental insight into the process of nucleocytoplasmic transport will help to dissect the role of defects in this process in ALS. This is exemplified by recent work, suggesting that the nuclear export signals (NES) of TDP-43 and FUS are not functional and that these proteins may leave the nucleus by passive diffusion. Retention to synthesized RNAs sequesters them inside the nucleus and limits cytoplasmic diffusion [41]. This would suggest that defects in active nuclear export of TDP-43 and FUS only play a minor role in ALS and helps to understand how these proteins accumulate in the cytoplasm in ALS.

## Conclusions

Intracellular trafficking defects observed in ALS range from accumulation or mislocalization of cell surface receptors or disturbed ER/Golgi trafficking to perturbations in motor proteins and the cytoskeleton. This review highlights that these defects result from changes in molecular and cellular processes that are often not isolated events but rather steps of a continued trafficking pathway. A particular phenotype, such as protein accumulation, may be explained by changes in several of the steps of the trafficking process. For example, cell surface receptor accumulation could result from altered Rab function, defects in motor proteins carrying this cargo, or from changes in the cytoskeleton. In addition, these defects may also indirectly affect other processes. For example, downregulation of Rabs can result in altered expression of their effectors. This may induce up- or downregulation of Rab proteins in complementary networks [176] and cause defects such as uncontrolled budding or fusion of vesicles [74, 110].

While evidence is accumulating that trafficking defects significantly contribute to motor neuron death and ALS, our understanding how trafficking is affected and how these changes lead to ALS and could eventually be counteracted to treat this disease is rather incomplete. This situation is exemplified by a large number of recent studies on C9ORF72 ALS/FTD. C9ORF72 repeat expansions are known to lead to reduced C9ORF72 expression which is thought to affect protein degradation. At the same time, decreased C9ORF72 expression may trigger hyperexcitability through effects on glutamate receptors and thereby induce neuronal death. While the precise contribution of C9ORF72, loss of function to motor neuron degeneration remains unclear, the pathogenic gain of function effects of C9ORF72 repeat expansions, such as the formation of RNA foci and DPRs, also targets intracellular trafficking, e.g., axonal transport and nucleocytoplasmic trafficking. Finally, C9ORF72 loss and gain of function mechanisms may interact. It has been suggested that while *C9ORF72*^−/−^ mice do not show overt neurodegeneration [89], reduced C9ORF72 activity could impair the clearance of DPRs and thereby enhance the effects of these toxic proteins [194]. Future work is needed that systematically dissects the downstream effects of C9ORF72 repeat expansions and other ALS mutations on different aspects of intracellular transport. High-resolution live imaging, humanized culture models, and manipulation strategies such as CRISPR/CAS to perform gene knockout or induce epitope tags to endogenously label proteins of interest should be part of the toolbox to further explore the contribution of trafficking defects in ALS.

Many open questions with respect to intracellular trafficking and ALS remain. For example, how do intracellular transport and protein aggregates interact and which are the functional consequences of this interaction. Protein aggregation is a pathological hallmark of ALS. TDP-43 aggregates are found in the majority of patients, while several other ALS-associated proteins are prone to aggregate, e.g., SOD1, FUS, and DPRs. Defects in intracellular trafficking are linked in several ways to disturbed protein homeostasis. First, in some cases, mutant proteins accumulate and start to form aggregates because of defects in transport, e.g., nucleocytoplasmic transport. Second, disrupted protein degradation due to perturbed intracellular trafficking may facilitate aggregate formation and stability. Third, aggregates can inhibit intracellular transport, e.g., axonal transport or nucleocytoplasmic trafficking. Thus, it is clear that intracellular trafficking contributes to aggregate formation, while aggregates disturb intracellular transport, but the precise molecular and functional details of these interactions remain to be dissected. While this review has focused on neuronal trafficking, non-neuronal cells such as glia cells may also contribute to ALS pathogenesis and trafficking defects. For example, activated microglia-conditioned medium induces neurite beading in cultured MNs via NMDA-R signalling. This signalling inhibits mitochondrial complex IV and a subsequent decline in ATP reduces fast axonal transport and accumulation of tubulin, neurofilament, kinesins, and dynein prior to MN death [168]. Therefore, further studies are needed to examine whether the intracellular trafficking defects observed in MNs are also at play in relevant populations of non-neuronal cells.

Finally, it is clear that at least some aspects of intracellular trafficking play a key role in ALS pathogenesis. Studies have begun to use these observations as starting points for designing novel therapeutic strategies for ALS (e.g., inhibition of p38 MAPK or HDAC6). Further insight into the role of defective intracellular transport will, therefore, undoubtedly provide further targets for the design of therapeutic interventions for ALS in the future.
